# Focal organizing pneumonia simulating lung malignancy: treated with prednisolone

**DOI:** 10.1002/rcr2.469

**Published:** 2019-08-05

**Authors:** Hemanth Kilaru, Mohd Vaseem Jalna, Satish Chandra Kilaru, Eshwar Chandra Nandury, Mohammed Zia Ur Rehman Khan

**Affiliations:** ^1^ Pulmonology Prathima Institute of Medical Sciences Karimnagar India; ^2^ Radiology Virinchi Hospitals Hyderabad India; ^3^ Pathology Virinchi Hospital Hyderabad India

**Keywords:** Focal, organizing pneumonia, prednisolone, treatment

## Abstract

Focal cryptogenic organizing pneumonia (FOP) is a localized form of cryptogenic organizing pneumonia (COP). It is an uncommon clinicopathological entity associated with non‐specific symptoms and radiographic findings simulating lung malignancy. Incidence of idiopathic FOP is not known and only reported as case series. Its treatment usually involved surgical resection. Here, we report a case of a 62‐year‐old female presenting with a history of dyspnoea, persistent paroxysms of dry cough, and low‐grade fever of three weeks duration with a solitary consolidation on imaging. Computed tomography‐guided biopsy showed an organizing pneumonia pattern. A therapeutic trial with prednisolone resulted in resolution without the need for surgical resection, without recurrence after follow‐up for 12 months.

## Introduction

Focal cryptogenic organizing pneumonia (FOP) is an uncommon clinicopathological entity associated with non‐specific symptoms and radiographic findings often simulating lung malignancy or pneumonia. It is defined histopathologically by the presence of buds of granulation tissue progressing from fibrin exudates to loose accumulations of collagen‐embedded fibroblasts in the distal air spaces. This pathological characteristic of intra‐alveolar fibrosis, resulting from organization of inflammatory exudates, reversibility with corticosteroids 1, emphasizes the need for early diagnosis and treatment, especially when it is focal with non‐specific features.

In view of few case reports highlighting the non‐surgical approach of treating FOP, we report on histopathologically confirmed case resolving with treatment with corticosteroids.

## Case Report

A 62‐year‐old female, never smoker, presented with gradually progressive dyspnoea, paroxysms of dry cough, low‐grade fever, and loss of appetite for three weeks. Prior to admission, she was treated with antibiotics for pneumonia and referred to our institute for evaluation of lung malignancy. Her past and family history was not contributory.

Her vitals were normal with peripheral capillary oxygen saturation of 96% at room air. The routine laboratory tests were unremarkable except for her erythrocyte sedimentation rate of 50 mm in the first hour, C‐reactive protein of 14 mg/dL, white blood cell count of 14,000/mm^3^.

**Figure 1:**
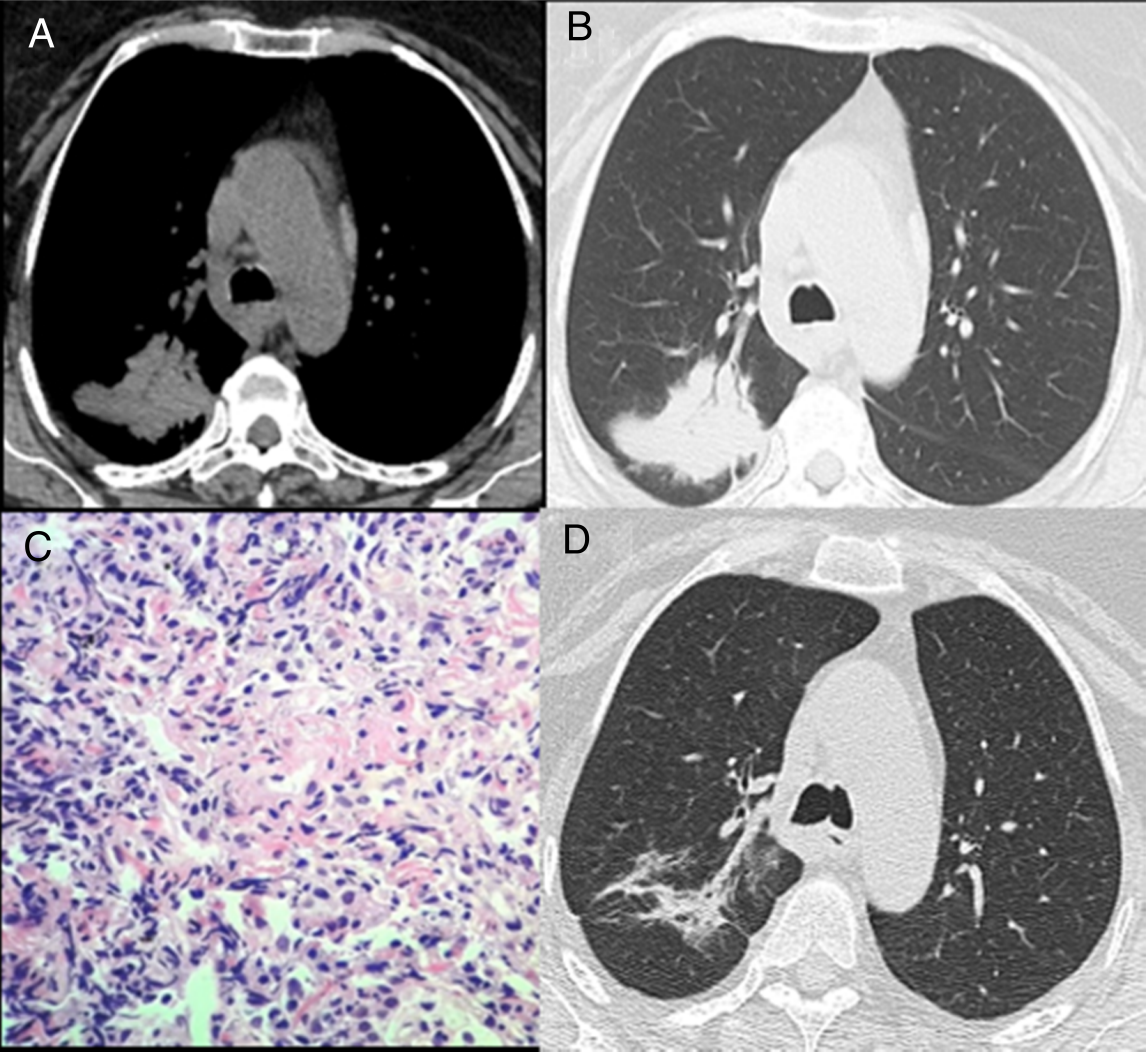
Computed tomography (CT) chest at admission: (A) mediastinal window and (B) lung window sections at the level of carina reveal a large lobulated soft tissue density lesion in the posterior segment of the right upper lobe with air bronchograms. (C) Histology haematoxylin and eosin section from CT‐guided biopsy of the right lung lesion at high power view (40×) shows fibroblastic foci with spindle‐shaped fibroblasts in hyaline matrix and inflammatory cells. (D) Follow‐up CT chest done six months post‐treatment reveals significant resolution of the lesion with a residual irregular atelectatic opacity.

High‐resolution computed tomography (CT) chest features were as shown in Fig. 1A, B, and CT‐guided biopsy done subsequently showed features suggestive of organizing pneumonia (OP) (Fig. 1C). She was started on prednisolone with a regimen of 0.75 mg/kg/daily for four weeks, followed by 0.5 mg/kg for four weeks, then 20 mg for four weeks, 10 mg for six weeks, and then 5 mg for six weeks. She showed signs of clinicoradiological improvement after four weeks of prednisolone. In a follow‐up visit, six months after her treatment was initiated, she was found to be asymptomatic and resolution of her lesion on CT chest (Fig. 1D). She remained asymptomatic and did not relapse after a year of follow‐up.

## Discussion

Cryptogenic OP (COP) is a clinicopathological syndrome characterized by rapid resolution with corticosteroids 2. It is classified as one of the idiopathic interstitial pneumonias. When OP manifests as solitary lesion, it is known as focal OP, which accounts for 13% of cases of OP and having 58% predilection for upper lobes 3. Delayed treatment can increase the risk of relapse. Though relapses are common (58%), a rapid clinical and radiographic improvement can be expected with corticosteroids 1, 2.

FOP can be asymptomatic or can have a subacute presentation with clinical manifestations of flu‐like illness with cough and progressive dyspnoea. Uncommon symptoms include haemoptysis, night sweats, mild arthralgia, and chest pain. Physical examination may reveal sparse lung crackles or can be unremarkable with non‐specific radiographic findings. The lesions are often located in upper lobes and may cavitate. The diagnosis of COP is usually delayed for 6–13 weeks either due to non‐specific manifestations or the patient does not improve with antibiotics given for a possible infectious pneumonia 1.

FOP is a rare presentation of COP, and studies regarding it are relatively limited. Furthermore, FOP frequently gives rise to diagnostic problems as it may present with imaging patterns that are similar to malignant lesions like bronchogenic carcinoma including positive results on contrast‐enhanced CT scan and positron‐emission tomography scanning. Hence, the diagnosis is usually made after surgical resections 3. Though bronchoalveolar lavage fluid may help in excluding other diagnoses or determining a cause of secondary COP, video‐assisted thoracoscopy and transbronchial lung biopsy may be considered in patients with suspected COP 1. Additionally, despite limited experience on the role of CT‐guided lung biopsy, it is another safe and affordable method that can be used to diagnose COP which obviates the need for unnecessary surgery 4. In our case, CT‐guided biopsy showed “OP‐pattern” and we opted for a trial of prednisolone, as proposed by Lazor et al., with follow‐up for three to four weeks, for clinicoradiological improvement, and later tailing off prednisolone over six months. Relapses can be managed with low doses of corticosteroids and short duration of treatment 1, 2. Majority of the studies available on the treatment of FOP advocate a surgical approach.

Owing to the rarity of its presentation and often simulating pneumonia or malignancy, even on imaging, FOP can cause a considerable diagnostic dilemma. We suggest that in patients with suspected FOP, a CT‐guided biopsy to confirm histopathological diagnosis and a trial with corticosteroids and clinicoradiological follow‐up for four weeks be considered which may obviate the necessity for a surgical procedure.

### Disclosure Statement

Appropriate written informed consent was obtained for publication of this case report and accompanying images.
